# Epidemiological and clinical characteristics, spirometric parameters and response to budesonide/formoterol in patients attending an asthma clinic: an experience in a developing country

**DOI:** 10.11604/pamj.2015.21.154.5404

**Published:** 2015-06-24

**Authors:** Hassan Imad, Ged Yasir

**Affiliations:** 1 Saud bin Abdulaziz University for Health Sciences, Department of Medicine 1443, King Abdulaziz Medical City, King Fahad National Guard Hospital, PO Box 22490, Riyadh 11426, Kingdom of Saudi Arabia; 2 Medical Officer, Department of Medicine, National Ribat University Hospital, Khartoum, Sudan

**Keywords:** Asthma, developing country, spirometry, budesonide/formoterol, therapy

## Abstract

**Introduction:**

This study aims at describing the epidemiological and clinical characteristics, severity, reversibility testing and response to treatment using simple spirometry in asthmatic patients attending a model specialized Asthma Care Center.

**Methods:**

Eligible subjects must have a suggestive clinical picture and confirmed by spirometry to have a 12% plus 200ml absolute increase in FEV1 either by reversibility testing or after a therapeutic trial with inhaled and/or oral steroid therapy. Budesonide-Formoterol Turbohaler was used for reversibility testing and for maintenance therapy with or without the addition of oral prednisolone.

**Results:**

One hundred and nineteen patients were eligible for the study. Age ranged between 10 -70 years. One hundred and thirteen patients (95.0%) had an FEV1 less than 80% of predicted. One hundred and five patients (88.2%) had reversibility testing of whom 72 (68.6%) had a significant reversibility. Sixty two patients (52.1%) were prescribed Budesonide-Formoterol Turbohaler only whilst 57 were prescribed both Budesonide-Formoterol Turbohaler and oral prednisolone. Patients were reviewed after a mean of 14.9 days (range 6.0-28.0). Seventy two patients (60.5%) had increased their FEV1 to more than 80% of their predicted value. By logistic regression analysis, predicted FEV1 at baseline was a significant negative predictor of a complete response.

**Conclusion:**

Most patients had abnormal spirometry with more than half having an FEV1 that is 60% or less of their predicted normal reading. Reversibility testing using Budesonide-Formoterol Turbohaler confirmed the fast onset of action of its Formoterol component and helped in cutting the cost of this test. The majority improved with treatment with 60% normalizing their spirometry highlighting the feasibility and applicability of specialized asthma care centers in resource-poor countries.

## Introduction

The global burden of Bronchial Asthma (BA) particularly in developing countries is on the increase [1, 2]. Approximately 300 million people worldwide have asthma, and its prevalence seems to increase by approximately 50% every ten years [1, 2]. BA is not a benign disease and has clearly had a serious impact on national health care systems expenditure in developed countries, patient's quality of life and more importantly has a noteworthy mortality risk [1–4]. International asthma guidelines emphasize the importance of a comprehensive asthma care delivery service [5]. All guidelines highlight the importance of proper diagnosis of BA, individualized therapeutic interventions based on assessment of its severity, patient education and regular follow-up. Despite the accessibility and availability of these guidelines on the World Wide Web, BA care remains suboptimal in many countries [1]. In Khartoum, the Capital of Sudan we established an Asthma Care Center aiming to provide a higher quality asthma care service. Structured care, patient education and spirometry were its pillars. All patients were prescribed and thoroughly educated on either breath-activated devices or metered dose inhalers administered via spacer devices. In this study we investigated the outcome of our intervention in the subgroup of patients who were prescribed Budesonide-Formoterol Turbohaler (BFT) (160 ug/4.5 ug). This investigation aims to describe the epidemiological characteristics and define by simple spirometry the severity, reversibility and spirometric response to treatment with BFT in a cohort of Sudanese patients with Bronchial Asthma. The clinical efficacy and cost-effectiveness of BFT in chronic asthma care is well documented [6, 7]. Inhaled Formoterol- a long acting beta 2 agonist with a fast onset of action and kinetics similar to inhaled salbutamol [8, 9] is used for reversibility testing. The rationale for its use as such are first to help relief patients of their symptoms before leaving the clinic, secondly as an opportunity to train patients on inhaler technique and possibly improve compliance as patients note the immediate effectiveness of their inhaler and thirdly to reduce the cost and the time it takes to do reversibility testing.

## Methods

### Study design

A retrospective chart review of patients with Bronchial Asthma. Patient's consent was not sought as this was a retrospective chart review study and there was no disclosure of confidential patient information.

### Setting

This study was undertaken in a dedicated Bronchial Asthma Care Center in Khartoum, Sudan. The center is manned by a Consultant Pulmonologist, Asthma Educator/Spirometry technician and a Clerk.

### Patients and medications

Patients attending the center with a clinical picture suggestive of Bronchial Asthma who were prescribed BFT and who were confirmed by spirometry to have a 12% increase plus 200 ml absolute increase in FEV1 after reversibility testing or after a steroid trial were eligible for inclusion in the study. A steroid trial was with inhaled steroid (budesonide 320 ug twice daily by turbohaler in Budesonide-Formoterol Turbohaler) for two weeks with or without oral prednisolone 30 mg per day for 5 days. Patients unable to do spirometry or have poor technique were excluded. Patients with a diagnosis of bronchiectasis, COPD or other respiratory diseases were also excluded. Patients were seen by one Pulmonologist. A detailed history is taken along a pre-specified and unified format detailing all the essential historical data pertaining to Bronchial Asthma. Patients were then seen by the Asthma Educator/Spirometry technician who performed baseline spirometry (hand-held spirometer -Spirodoc-GIMA) and educated the patients on their illness and their inhaler technique. Spirometry was performed with the patient sitting. The first two puffs of their BFT were then administered and spirometry repeated 15 minutes later. Patients must be able to have three attempts at spirometry with variations of less than 0.1 liter in FEV1 between the two best readings for a reliable trace. Most patients were requested to re-attend clinic in 2 weeks for clinical reassessment and spirometry. However, those attending up to 4 weeks post first visit were included. As normative spirometric data for Sudanese subjects are not available and these are generally believed to be 10 percent lower than those of Caucasian, Caucasian FEV1 data corrected by deducting 10% were utilized. Data was entered on a Minitab 15 spreadsheet for analysis. Simple descriptive statistical tests were used together with the 2-Sample t-test, Chi square test and logistic regression analysis as appropriate to test for significance and associations. A p-value of less than 0.05 is considered significant and the actual value quoted.

## Results

One hundred and nineteen patients were eligible for the study. Sixty two were male and 57 were female with an average age of 30.5 (range 10-70) and 30.3 (range 10-68) years respectively. Mean body mass index was 23.2 (range 11.8-33.2) for males and 27.4 (range 14.3-44.1) for females. A BMI of more than 30 was more frequent in females (20) versus (6) in males (Chi Square Test p value= 0.001). Ninety patients (75.6%) were previously diagnosed as having bronchial asthma. Mean duration of asthma for male patients was 156.9 months (range 6-720 months) and for females was 191.4 months (range 1.2-600 months). Twenty four of the known asthmatic patients (26.7%) have never been prescribed inhaler therapy before. The mean duration of asthma for this group was 156 months (range 1.2-540 months). Family history in first degree relatives for asthma was positive in 73.9% of patients. Of 66 patients who could describe their inhalers, thirty nine (59.1%) were on a steroid inhaler. Twenty patients (16.9%) were active or previous smokers. Eleven patients reported an allergic reaction to food of whom 6 implicated Aubergines as a precipitant Table 1.

**Table 1:** Demographic and clinical data

Parameter	Number (Percent)	Male	Female
**Gender**	119	62	57
**Age-Years (mean, range)**		30.5 (10-70)	30.3 (10-68)
**BMI (mean, range)**		23.2 (11.8-33.2)	27.4 (14.3-44.1)
**Smoking**	20 (16.8%)	18	2
**PH of B Asthma**	90 (75.6%)	48	42
**Duration of B Asthma-Months (mean, range)**		156.9 (6.0-720.0)	191.4 (1.2-600.0)
**Cough**	112 (94.1%)		
**Sputum^a^**	80 (72.1%)		
**SOB**	112 (94.1%)		
**Wheeze^b^**	98 (85.2%)		
**GERD^c^**	40 (35.7%)		
**Allergic Rhinitis/Sinusitis/Postnasal Drip^d^**	64 (54.7%)		
**Family History of Asthma**	88 (73.9%)		
**Previous Use of Inhalers**	66 (73.3%)		
**Previous Use of Steroid Inhalers**	39 (59.1%)		

Missing data:

a : 7 patients

b : 4 patients

c : 7 patients

d : 2 patients

### Symptoms

Cough and shortness of breath were the commonest symptoms and were reported by 112 patients (94.1%). Wheeze was reported by 98 patients (85.2%). Eighty patients (72.1%) had productive cough.

### Gastro-esophageal and Upper Respiratory Symptoms

Forty patients (35.7%) admitted to have frequent heartburn and 64 patients (54.7%) reported symptoms suggestive of allergic rhinitis and/or postnasal drip. Those with gastro-esophageal reflux disease (GERD) symptoms had a lower percent predicted FEV1 than those without, but this did not reach statistical significance: 52.0% vs 57.7% (Two-sample t-test p= 0.10). Percent FEV1 readings were comparable in those with and those without upper respiratory symptoms: 54.8% versus 56.0% (Two-sample t-test p= 0.72).

### Wheeze on examination

On auscultation 84 (70.6%) patients had audible wheeze.

### Baseline FEV1 and Severity Classification (Table 2)

**Table 2:** Spirometric data

Parameter	Number/Percentage	Male	Female
**Baseline FEV1 (mean, range)**	119	1.8 (0.6-3.2)	1.5 (0.5-2.4)
**Baseline %FEV1 of Predicted (mean, range)**	119	52.9 (18.7-87.7)	57.9 (14.5-85.6)
**% Baseline FEV1 less than 80% of Predicted**	113 (95.0%)	60	53
**% Baseline FEV1 less than 60% of Predicted**	68 (57.1%)	39	29
**Absolute FEV1 change post Formoterol (ml) (mean, range)**	105 (88.2%)	365.5 (10.0-1130.0)	313.0 (-100.0-710.0)
**%FEV1 Change after Reversibility Testing(mean, range)**	105 (88.2%)	23.0 (0.7-71.2)	23.1 (-11.7-71.8)
**Patients with Significant Reversibility**	72 (68.6%)	39	33
**Absolute Change in FEV1 in those with Significant Reversibility (mean, range)**	72 (68.6%)	474.4 (200.0-1130.0)	405.2 (210.0-710.0)
**Percentage Change in FEV1 in those with Significant Reversibility (mean, range)**	72 (68.6%)	29.8 (12.1-71.2)	30.4 (12.1-71.8)
**Absolute FEV1 after Treatment (L) (mean, range)**	119	2.6 (1.1-4.8)	2.2 (1.0-2.9)
**%FEV1 Change after Treatment (mean, range)**	119	65.4 (-1.7-300.0)	59.7 (11.7-274.0)
**%FEV1 of Predicted after Treatment (mean, range)**	119	79.1 (34.3-126.5)	85.7 (41.3-124.6)
**% FEV1 more than 80% of Predicted post- Treatment**	72 (60.5%)	37	35

Mean FEV1 at baseline was 1.8 L (range 0.6-3.2L) for males and 1.5 L (range 0.5-2.4L) for females. Expressed as a percent of the predicted normal values, mean FEV1 in males was 52.9% (range 18.7-87.7%) and for females was 57.9% (range 14.5-85.6%). Wheezy patients had a significantly lower predicted FEV1 than those with no audible wheeze: 50.4% versus 67.1% (Two sample t test p value< 0.0001). One hundred and thirteen patients (95.0%) had an FEV1 less than 80% of predicted and 68 (57.1%) had a reading that is less than 60% of predicted. Eight (11.8%) of the latter group did not have audible wheeze on auscultation.

### BFT reversibility testing (Table 2, Figure 1)

**Figure 1:**
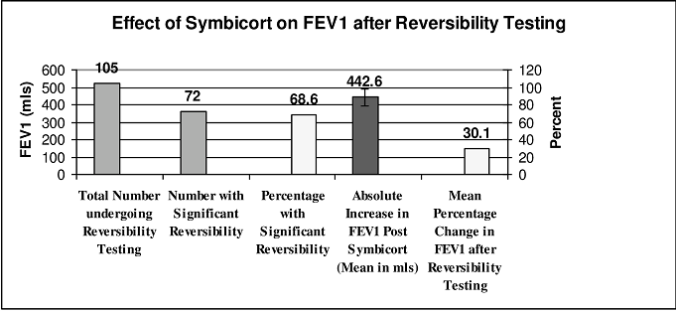
Effect of budesonide/formoterol on fev1 after reversibility testing

One hundred and five patients (88.2%) had reversibility testing of whom 72 (68.6%) had a significant change of 12% plus 200 ml increase in FEV1. The average percent increase in FEV1 in this group was 30.1% (range 12.1-71.8%) with a mean absolute increase of 442.6 mls (range 200-1130 mls). All of those who did not have an initial reversibility testing (14) and those who did not attain a significant change after Formoterol (33) did achieve a 12% plus 200 ml increase following their course of treatment. Mean percentage increase in FEV1 was 65.5% (range 13.4-297.0%) with an absolute mean increase of 746.6 mls (range 230.0-2200mls). Prescribed Medications and Symptomatic Response to Therapy Sixty two patients (52.1%) were prescribed BFT only whilst 57 were prescribed both BFT and oral prednisolone. Of the latter group, 48 patients (84.2%) had an FEV1 of less than 60% of predicted. Patients were reviewed after a mean of 14.9 days (range 6.0-28.0). On their second visit, 118 reported that their symptoms improved (99.2%) of whom 74 (62.2%) said their symptoms have disappeared or much improved.

**Figure 2:**
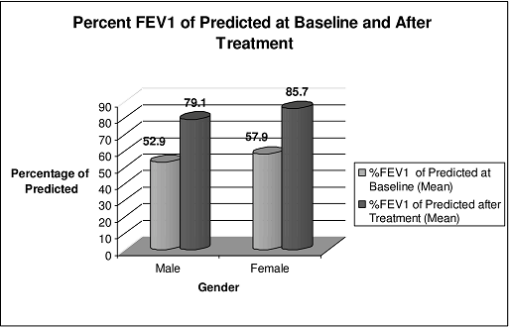
Percent fev1 of predicted at baseline and after treatment

**Figure 3:**
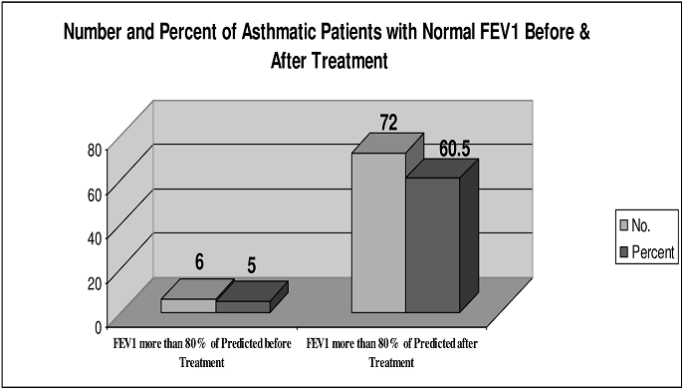
Number and percent of asthmatic patients with normal fev1 before and after treatment

Spirometric Response to Therapy (Table 2, Figure 2 and Figure 3) The average FEV1 reading post treatment was 2.6 L in males (range 1.1-4.8) and 2.2 L in females (range 1.0-2.9). The respective average percent increase in FEV1 were 65.4% (-1.7-300%) and 59.7% (range 11.7-274%). Expressed as a percent of their predicted normal values, mean FEV1 in males has increased from 52.9% to 79.1% and for females from 57.9% to 85.7%. Mean percentage FEV1 increase in the BFT group was 33.7% (range -1.74-167.9) compared to 94.2% (range 6.9-300) in the BFT plus oral prednisolone group (two-sample t-test p < 0.0001). Seventy two patients (60.5%) had increased their FEV1 to more than 80% of their predicted value i.e. normalized their spirometry.

### Predictors of Complete Response (Table 3)

**Table 3:** Complete and incomplete responders comparison table

	Complete Responders	Incomplete Responders	P value
No	72 (60.5%)	47 (39.5%)	
Age- years (mean, range)	30.3 (10.0-70.0)	30.6 (10.0-60.0)	0.38^a^
Duration of Asthma (days) (mean, range)	145.2 (1.2-600)	206.2 (6.0-720)	0.19^a^
Baseline % FEV1 of Predicted (mean, range)	62.6 (24.1-87.7)	44.1 (14.5-76.5)	<0.0001^a^
% Change in FEV1 after Reversibility Testing (mean, range)	21.6 (-6.33-71.2)	25.0 (-11.7-71.8)	0.97^a^
% Change in FEV1 after Treatment (mean, range)	62.5 (8.0-300.0)	63.0 (-1.7-292.5)	0.97^b^

a : Logistic Regression Analysis

b : 2-Sample t-test

The mean percent increase in FEV1 in those who normalized their FEV1 as opposed to those who did not were 62.5% (range 13.4-300) and 63.0% (range -1.7-292.5) respectively (Two-sample t-test p = 0.82). By logistic regression analysis, predicted FEV1 at baseline was a significant negative predictor of a complete response with age, duration of asthma and initial FEV1 change to reversibility testing having no such correlation. Forty six patients who were prescribed BFT normalized their spirometry compared to 26 patients who were prescribed BFT plus oral prednisolone (Chi Square test p= 0.001). Mean percent FEV1 of predicted at baseline was 66.6% for the former and 43.0% for the latter (Two-sample t-test p <0.0001).

## Discussion

An initial striking feature in this study is the extremely low baseline spirometric readings of this cohort of patients. This highlights the deficiency of care this cohort of patients was receiving. Anti-inflammatory therapy is the mainstay of treatment of Bronchial Asthma [5]. More than a quarter of the known asthmatics were not on inhaler therapy and more than a third were not on steroid inhalers. Furthermore, what we have very frequently observed is the very poor inhaler technique in these patients. This has previously been documented in a study in the main respiratory hospital in Khartoum where 82% of patients had poor inhaler technique [10]. This finding emphasizes the importance of patient education on their inhaler devices. This latter service has been one of the major objectives and strength in our asthma care center service. The use of BFT in our cohort was in accordance with Global Initiative for Asthma (GINA) guideline recommendation as the majority had relatively severe and highly symptomatic asthma [5].

The presence of audible wheeze on examination was clearly a marker of severity as these patients on the whole, had a significantly lower predicted FEV1 readings. However, almost a third of this cohort of asthmatic patients had no wheeze on examination including no less than 10% of those with the more severe spirometry readings. Spirometric assessment of suspected patients with Bronchial Asthma is thus extremely vital for quality care. Obesity is seen in a proportion of our patients specially females. As is known, obesity is a risk factor for asthma and is also associated with poor asthma control and needs to be properly managed by dieting and weight reduction [11]. Almost a third of our patients admitted to having frequent GERD symptoms and more than a half to upper respiratory symptoms. FEV1 in those with GERD were clearly worse than in those without implying a causal or contributory effect and underscoring the importance of inquiring about and managing GERD symptoms [12, 13]. All our patients with GERD were prescribed proton-pump inhibitors and were given life style modification advice. Of interest is the reported food allergy to Aubergine (Black Egg Plant) that is seen in our cohort. This seems to be the commonest “food allergen” that is implicated in adult Sudanese patients with asthma. However, there is a debate on whether this represents a true allergic response to constituent protein products or a non-allergic pharmacologic reaction to histamine-like and other components of the eggplant [14, 15]. These reactions are reported to be more prevalent in atopic individuals [14, 15].

Almost three-quarters of our patients had significant increases in FEV1 after reversibility testing. The use of the prescribed medication (BFT) for reversibility testing assisted us in realizing many goals of our service. The need of using salbutamol MDI via spacer or nebulizer for testing was no longer required. This meant that we were able to save on reagent cost (Salbutamol MDI or nebulizer solution) and spacer devices or nebulizer machine use (unit price plus sterilizing between patients). We had more time to educate these patients on their inhaler device use instead of training on MDI via spacer etc. use for reversibility testing. We were very pleased to see very symptomatic and breathless patients feeling already improved following the use of their new medication and before leaving clinic. We hoped that this will translate into better patient compliance.

The outcome of our interventions was similarly very satisfactory. Almost all of our patients reported improvements in their symptoms and a good proportion had become asymptomatic or remarked that they were much improved. This was mirrored by the large improvements in the patient's FEV1. More than 60% of patients normalized their FEV1. The most important (negative) predictor of FEV1 normalization was the baseline reading. Patients with very poor spirometric figures were less likely to achieve their target normal reading. Patients with incomplete responses had, albeit non-significant difference, a longer duration of Bronchial Asthma. This emphasizes the importance of early diagnosis and proper asthma care interventions [16, 17]. Addition of systemic steroids to BFT in those with FEV1 readings below 60% of predicted was prudent as it has clearly lead to a more satisfactory, significant and worthwhile improvement in spirometry. More patients on BFT alone normalized their FEV1 compared to those on BFT and prednisolone. This is clearly a reflection of the lower baseline FEV1 readings in the latter group and underlines the need for longer courses of treatment.

## Conclusion

In summary, our objectives of providing a satisfactory dedicated service to asthma patients were fulfilled. The use of BFT for an average of two-weeks with or without oral prednisolone was effective in improving the symptoms in almost all of our patients who were prescribed this inhaler. Furthermore, a good proportion (60%) normalized their spirometry. Those with very poor FEV1 readings at the outset (more than 50% of our cohort) were less likely to normalize their spirometry. The magnitude of improvement in FEV1 was appreciable in all grades of severity provided that oral prednisolone is added to BFT in those with FEV1 of less than 60% of predicted. The importance of routine spirometry in Bronchial Asthma care particularly in severity assessment and choice of therapy cannot be overemphasized.
